# Supplementary material for: Identification of photoperiod-induced specific miRNAs in the adrenal glands of Sunite sheep (Ovis aries)
Source: Front Vet Sci. 2022 Jul 22;9:888207. doi: 10.3389/fvets.2022.888207 (PMC9354845; doi:10.3389/fvets.2022.888207)
Supplement: Supplementary file 1 [file Data_Sheet_1.ZIP › Supplementary file/miRDeep2 file.zip!/miRDeep2 file/Novel_Structure/SPLP42A3/Novel_125.pdf]

Provisional ID	:	NC_019460.2_10510
Score total	:	2.1
Score for star read(s)	:	-1.3
Score for read counts	:	0
Score for mfe	:	1.8
Score for randfold	:	1.6
Score for cons. seed	:	
Total read count	:	34
Mature read count	:	34
Loop read count	:	0
Star read count	:	0

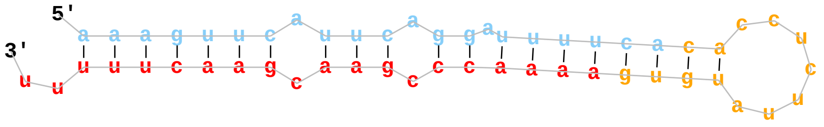

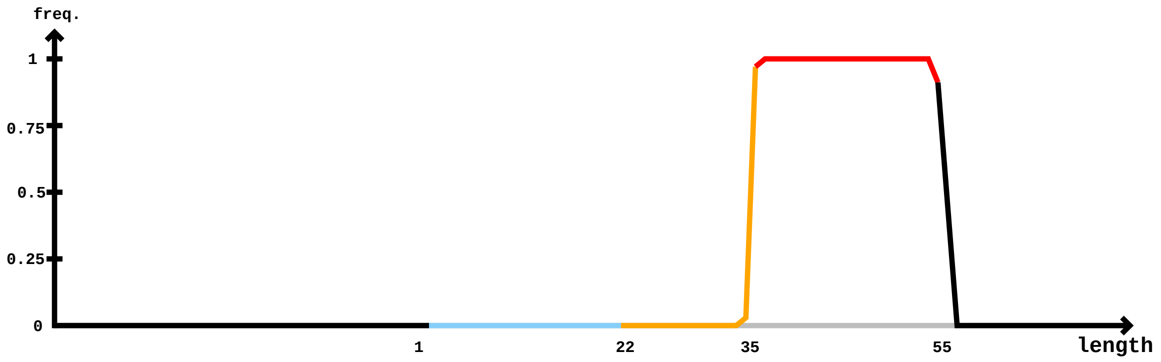


```
                                                        Star                          Mature
5'- gcuaguagaggggaaagacaaagauuggguuggucaaaaaguucauucaggauuuucacaccucuuaugugaaaacccgaacgaacuuuuucaccaacccaauacaaagu   -3'    exp
    .......................(((((((((((.(((((((((.(((.((.((((((((.......)))))))))).))).))))))))).))))))))))).......     reads      mm         sample
    ......................................................................gaaaacccgaacgaacuuuu....................     1          0          seq
    .......................................................................aaaacccgaacgaacuuuu....................     2          0          seq
    .......................................................................aaaacccgaacgaacuuuuu...................     28         0          seq
    .......................................................................aaaacccgaacgaacuuuuC...................     1          1          seq
    .......................................................................aaaaccAgaacgaacuuuuu...................     1          1          seq
    ........................................................................aaacccgaacgaacuuuuu...................     1          0          seq
```
